# popSTR2 enables clinical and population-scale genotyping of microsatellites

**DOI:** 10.1093/bioinformatics/btz913

**Published:** 2019-12-05

**Authors:** Snædis Kristmundsdottir, Hannes P Eggertsson, Gudny A Arnadottir, Bjarni V Halldorsson

**Affiliations:** 1 deCODE genetics/Amgen, Reykjavík 102, Iceland; 2 School of Science and Engineering, Reykjavík University, Reykjavík 102, Iceland

## Abstract

**Summary:**

popSTR2 is an update and augmentation of our previous work ‘popSTR: a population-based microsatellite genotyper’. To make genotyping sensitive to inter-sample differences, we supply a kernel to estimate sample-specific slippage rates. For clinical sequencing purposes, a panel of known pathogenic repeat expansions is provided along with a script that scans and flags for manual inspection markers indicative of a pathogenic expansion. Like its predecessor, popSTR2 allows for joint genotyping of samples at a population scale. We now provide a binning method that makes the microsatellite genotypes more amenable to analysis within standard association pipelines and can increase association power.

**Availability and implementation:**

https://github.com/DecodeGenetics/popSTR.

**Supplementary information:**

Supplementary data are available at *Bioinformatics* online.

## 1 Introduction

Microsatellites, a.k.a. short tandem repeats (STRs), are tandem repeats with repeat motif lengths between one and six base pairs. They are one of the most frequent types of variation in the human genome, surpassed only by single nucleotide polymorphisms (SNPs) and indels and have a mutation rate estimated to be three to five orders of magnitude higher than for other types of genetic variation (Jónsson *et al.*, 2017; Sun *et al.*, 2012). Genotyping microsatellites from whole-genome sequence (WGS) data is challenging since they are highly polymorphic and library preparation methods may modify the true number of repeats in the sequence (Gymrek *et al.*, 2012). WGS-based association and clinical analysis commonly do not consider microsatellites, partially due to a lack of tools capable of analyzing them.

Tandem repeat expansions occur when microsatellites expand beyond a certain length threshold, making them unstable and thus more likely to expand further. A number of repeat expansions are known to be disease-causing (Gatchel and Zoghbi, 2005) and an increase in the use of WGS-technologies for genetic diagnostics has created a need for fast estimation of the repeat number at disease-associated loci.

Here, we present extensions to our previously published software popSTR and improvements of its previous implementation, both with respect to runtime and accuracy. We increased our expansion detection sensitivity, updated our sample specific slippage estimation kernel, reduced the dimensions of our logistic regression model and updated external libraries to decrease I/O time and handle both BAM and CRAM files. We further created a panel of known repeat expansion markers and a pipeline to determine at each loci whether read support for a pathogenic expansion is present. Last, we provide a method to bin genotypes into user specified bins to increase power of downstream association analysis. By combining this set of functionalities, we hope to make popSTR2 applicable in a wide range of situations. Both when analyzing large cohorts to make population inferences and disease associations as well as analyzing small sets or single samples in a clinical context.

## 2 Materials and methods

**Fig. 1.**
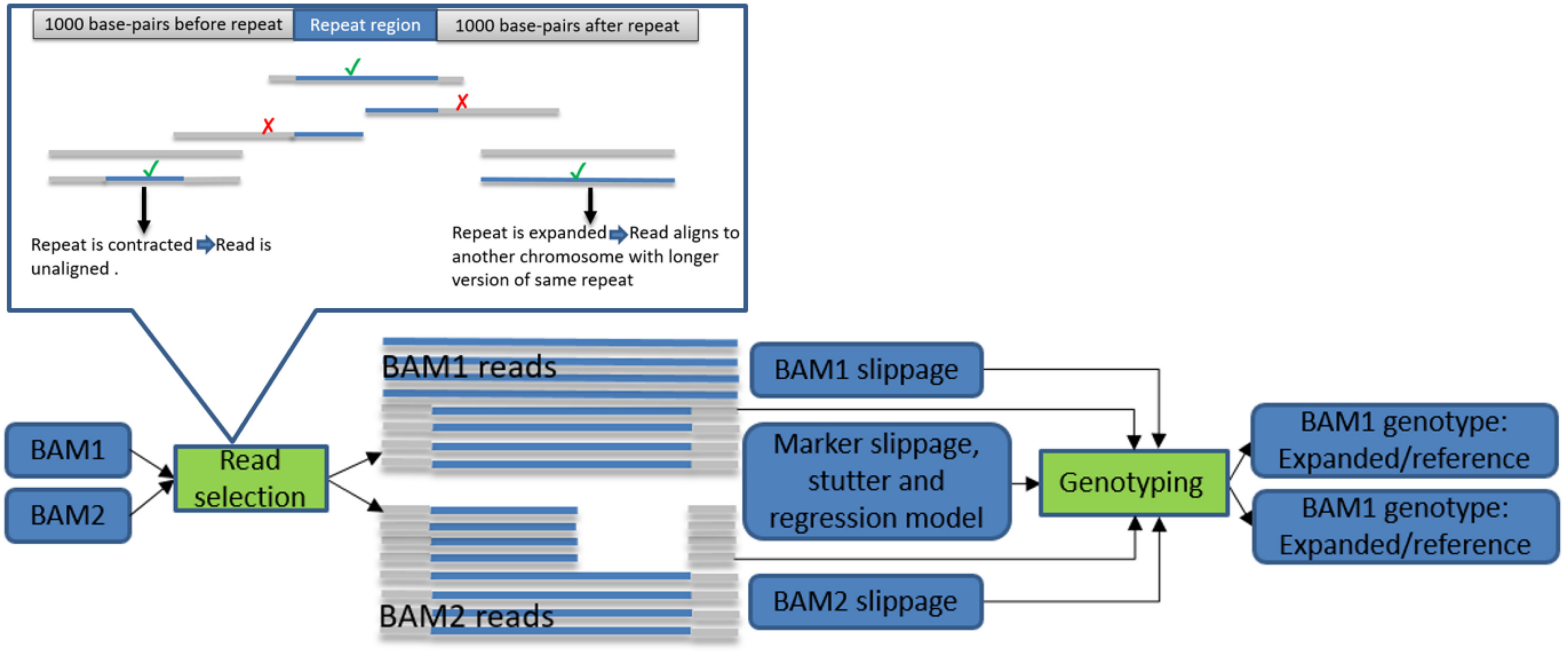
Results of read selection are passed into genotyping model along with sample and marker-specific parameters

Figure 1 gives a high level description of the algorithm’s workflow, a more detailed description is given in Supplementary Section S1.1 and a full description is given in Kristmundsdóttir *et al.* (2017). To summarize, we start by computing various quality-indicating attributes for all reads encompassing each of the microsatellites being considered, i.e. overlapping its coordinates and containing repeats of the relevant motif. We also look for repeats in unaligned reads with mates aligned close to the repeat region. An update of our read selection step is to also look for repeats of the relevant motif in reads aligned to longer repeats of the same motif in other locations of the genome that have mates aligned close to the repeat region. This can happen when a repeat has expanded considerably and the read reporting it is thus highly divergent from the reference sequence. After the set of informative reads has been created, the algorithm iterates between genotyping and assigning to each read a probability of reporting a true allele. Since this type of iterative parameter estimation is time and resource intensive, we supply a kernel of reliable markers to efficiently estimate these parameters. For details on kernel construction see Supplementary Section S1.2. We replaced the SeqAn BAM I/O module (Reinert *et al.*, 2017) with the one from htslib (Li *et al.*, 2009; https://github.com/DecodeGenetics/SeqAnHTS). The update provides CRAM file support, decreases I/O demands and runtime. Algorithmic improvements reduced runtime from 11.25 to 2.17 CPU hours/million markers per sample. See Supplementary Table S1 for a breakdown of our runtime analysis.

### 2.1 Application to population-based genotyping

Useful reads and their attributes are used along with marker and sample specific parameters to perform genotyping. The marker-specific parameters can be estimated by popSTR2, but we also provide a default set of parameters. By default we require 20 samples for the parameters to be estimated since estimation with fewer samples would not yield reliable results. The sample-specific slippage parameter is estimated using a kernel of reliable markers described above and supplied with the software. Our genotyping model (Supplementary Equation S1 in Supplementary Material) computes the likelihood of observing a read, *r*, given genotypes *A* and *B* and selects the genotype pair that maximizes this likelihood over the set of reads being considered. The model previously assumed constant probabilities of adding and removing repeats across all markers, fixing $a_{r}^{A}$ in Supplementary Equation S1 from Supplementary Material to 0.85 if whole repeats were removed and consequently to 0.15 if whole repeats were added. It has however been shown that microsatellites have very different mutation profiles depending on their various properties, e.g. repeat motif, repeat purity, reference allele length, etc. (Brinkmann *et al.*, 2002). To reflect this we have replaced the hard coded values with marker-specific estimates, computed as follows. Assuming that we know which reads result from whole motif slippage events, we can estimate the fraction of slippage events that added whole repeats at microsatellite *i*:

(1) $$p_{i}^{u}=\frac{\sum_{r\in R_{i}^{!u}}p_{i}(r)}{\sum_{r\in R_{i}^{!}}p_{i}(r)}$$

where $R_{i}^{!u}$ is the set of reads at microsatellite *i*, considered to be results of slippage events that add whole motifs and $R_{i}^{!}$ is the set of all reads at microsatellite *i* reporting whole motif slippage events, regardless of their direction. The probability of removing repeats is then trivially computed as $p_{i}^{d}=1-p_{i}^{u}$.

Our previous version created one output file per sample and computed nine attributes from each read used for genotyping. Due to increased data quality and consistency we were able to reduce the number of attributes to six, which simplified and sped up the logistic regression analysis. To make population scale inferences and genotyping easier we now write one output file per marker, i.e. all alleles discovered in a population accessible in the same file.

Association pipelines commonly assume biallelic variants or multi allelic variants where only a single allele is tested for association with a phenotype, rather than associating a subset of the alleles with it (Gudbjartsson *et al.*, 2015; Purcell *et al.*, 2007). This is not optimal for microsatellites where alleles above or below a certain length threshold may be pathogenic (Lee and McMurray, 2014). In an effort to increase association power we provide binSTR, a software for grouping alleles as a preprocessing step for association analysis. To allow for various patterns of allele groups, binSTR enables not only binarizing but also binning into a user determined number of groups where each group is defined by a list of allele indices passed as a parameter.

### 2.2 Application to clinical genetics

We have, through literature review, assembled a panel containing 31 STR markers, each associated with a disease or syndrome when the number of repeats passes a certain threshold, hereafter referred to as pathogenicity threshold. We provide a script which reports which of these markers, if any, contain evidence of a repeat expansion. The script runs the read selection step described above to scan a given BAM file at all panel locations and extracts for each of them all reads containing information on the number of repeats present. Expanded alleles have often undergone a dramatic increase in length, decreasing the odds of finding informative reads supporting them. Genotyping models assuming equal probabilities of drawing reads from each haplotype are thus not reliable in these cases. To account for this, our script scans the informative reads for any repeat tracts longer than the given threshold for each marker and flags locations harboring such reads for further manual inspection. Since many of the pathogenicity thresholds exceed the current read lengths by a considerable number of base pairs the scripts also counts and reports all fully repetitive reads, i.e. reads containing only repeats of the relevant motif. See Supplementary Table S4 for a table summarizing the markers included in the panel along with a pathogenicity threshold for each of them. As the set of pathogenic variants and our understanding of them grows the panel can easily be extended and thresholds for existing markers updated.

## 3 Experiments

We compared popSTR2 to HipSTR (Willems *et al.*, 2017), a commonly used microsatellite genotyper on chr21 of the CEU trio consisting of NA12878, NA12891 and NA12892 and on chr21 of 10 trios sequenced at deCODE genetics.

The runtime reduction was 40% for the CEU trio and 26% for the deCODE trios. To compare the accuracy of these two methods we extracted markers where both methods had high confidence genotypes for all members of at least one trio and at least one trio member had a non-homozygous-reference genotype and recorded the number of trios where the offspring genotype did not match the parental ones. The deCODE trios had slightly more accurate genotypes from popSTR2 than HipSTR (99.8% versus 99.6%) but for the CEU trio hipSTR had a single trio inconsistency in 250 markers while popSTR had 2. For a more detailed comparison of these runs see Supplementary Table S3. To examine the sensitivity of our expansion detection script we ran it on ten samples with a known expanded allele in the 3′-flanking region of the DMPK gene which causes myotonic dystrophy 1 when exceeding 50 copies (Musova *et al.*, 2009) and ten healthy control samples. The expanded samples were sequenced for clinical sequencing analysis at deCODE genetics and the healthy ones as parts of various other projects, also at deCODE genetics. The script flagged the DMPK locus in all expanded individuals and none of the control samples.

Last, we genotyped 49 962 Icelandic samples to examine the allelic spectrum of this repeat in the Icelandic population. The resulting distribution was in concordance with ones previously published for European populations with a bimodal distribution consisting of a peak at 5 repeats and another one between 11 and 13 repeats (Dean *et al.*, 2006; Magaña *et al.*, 2011) (see Supplementary Fig. S1).

## 4 Conclusion

We updated the microsatellite genotyper popSTR to decrease runtime and increase genotype quality and accuracy. This was done by replacing external libraries, re-training the data provided with the software and decreasing the number of variables in our logistic regression analysis. To expand the application range we extended the software to provide both a clinical sequencing analysis script for quickly estimating expansion status at known disease loci and a binning software for grouping genotypes by allele length range before performing disease association on them. It is our hope that these updates and extensions will make popSTR2 applicable in a broader spectrum of situations, i.e. for single sample clinical sequencing analysis as well as large scale association efforts. Analysis methods (Dashnow *et al.*, 2018; Dolzhenko *et al.*, 2017; Tang *et al.*, 2017; Tankard *et al.*, 2018) sensitive to detecting expanded repeats are not explicitly intended for population scale analysis of STRs at a genome wide scale. Conversely, other methods which aim at population and genome scale analysis (Gymrek *et al.*, 2012; Willems *et al.*, 2017) do not focus on and reporting of expanded repeats. GangSTR (Mousavi *et al.*, 2019) is, to our knowledge, the only method intended to perform accurate genotyping of both short and expanded microsatellites. It however does not mark known pathogenic variants in its output nor flags those expansions passing pathogenicity thresholds. By supplying a panel of known expansions along with an easily executable and fast script to flag potentially expanded repeats for further manual inspection we aim to direct users to the correct putative expansion as quickly as possible.

*Conflict of Interest*: none declared.

## Supplementary Material

btz913_Supplementary_Data
