# Supplementary material for: popSTR2 enables clinical and population-scale genotyping of microsatellites
Source: Bioinformatics. 2019 Dec 5;36(7):2269–71. doi: 10.1093/bioinformatics/btz913 (PMC7141861; doi:10.1093/bioinformatics/btz913)
Supplement: btz913_Supplementary_Data [file btz913_supplementary_data.pdf]

1 Supplementary materials

1.1 Algorithm

The algorithm takes a BAM/CRAM file and a list of microsatellite intervals to determine a set of reads informative of a sample's genotype. It considers reads encompassing each interval and reads with mates close to the repeat region that are either aligned to longer repeats of the same motif or could not be aligned at all. These misaligned and unaligned reads are considered to retrieve heavily contracted or expanded alleles that are highly divergent from the reference, see Figure 1. For each chosen read, a set of attributes, used to estimate read reliability, are computed and used as parameters when training logistic regression models for each marker. After the read-selection step, the algorithm iterates until convergence between genotyping according to latest parameter estimations and updating parameters using the latest genotypes. Since estimating parameters with few samples is not likely to yield reliable results, by default, we require 20 samples for the parameters to be estimated. A set of default parameters is supplied with the software for analysis of smaller sample sets. Comparing an allele reported by a read to a genotype, allows the algorithm to assign a label to the read. These labels, along with the read attributes, enable training of a per marker logistic regression model that assigns to each read, a probability $p_i(r)$ of reporting a true germline allele. The observed sequence may differ from the germline variant due to somatic mutations and sequencing problems which cause the addition or removal of full and partial motifs. The labels allow us to estimate marker-specific slippage and stutter rates S_i, t_i representing the probability of adding or removing a full or a partial motif, respectively. When determining the most likely genotype (A, B) given a read r, we use i as a marker identifier and j as a sample identifier in the following model.

$$
\begin{aligned}
L(r|A,B) = p_i(r) \cdot & \\
& \left(\frac{1}{2} \cdot pois(x_r^k(A); S_{ij}) \cdot geom(x_r^d(A); t_i) \cdot a_r^A \right. \\
+ \frac{1}{2} \cdot pois(x_r^k(B); S_{ij}) & \left. \cdot geom(x_r^d(B); t_i) \cdot a_r^B \right) + \frac{1 - p_i(r)}{n^i}
\end{aligned}
\tag{1}
$$

We let n^i be the number of alleles present in the population for microsatellite i. With probability $1 - p_i(r)$ the read being considered is an error read, in which case we assume that each allele is equally likely. We compute $x_r(A)$ as the number of slippage events needed to explain r with A. To separate whole and partial motif slippage events, we split $x_r(A)$ into its integer and decimal portions. We let $x_r^k(A)$ denote the integer portion which follows a Poisson distribution with $\lambda = S_{ij}$, a combination of the marker and sample-specific slippage rate estimates. $x_r^d(A)$ denotes the decimal portion of $x_r(A)$ which follows a geometric distribution with parameter t_i. We use Equation 1 in 2.1 of the main text to estimate and represent the different probabilities at each marker of adding and removing motifs. The result of this estimation is then used as a_r^A and a_r^B in Equation 1. Last, we select the genotype pair that maximizes the likelihood function in Equation 1 over the set of reads, R, being considered. Figure 1 in the main text shows a schematic view of the workflow described above.

1.2 Kernel construction

Our kernel was selected by genotyping all microsatellites on chr21 in a set of 6086 trios and choosing markers with heterozygous transmission rates between 30 and 70% and Mendelian error rates below 0.1% which resulted in 8779 markers. To verify that this subset accurately represented the entire marker set, we examined whether kernel markers were more likely to have larger repeat units and found that this was not the case, see Supplementary table 2. All our kernel training, parameter training and development was performed using BAM files aligned with the BWA-MEM aligner Li (2013) and generated using the sequencing processing pipeline described in Jónsson *et al.* (2017).

1.3 Repeat list construction

We created our list of repeat coordinates by running the Tandem repeats finder(trf) Benson (1999) on GRCh38 for each chromosome and retaining resulting repeats with motif lengths between one and six base pairs, (command: ./trf409.linux64 chr$num.fa 2 7 7 80 10 22 7 -d -h -ngs > trf.$num).

References

Benson, G. (1999). Tandem repeats finder: a program to analyze dna sequences. *Nucleic acids research*, **27**(2), 573–580.

Jónsson, H., Sulem, P., Kehr, B., Kristmundsdottir, S., Zink, F., Hjartarson, E., Hardarson, M. T., Hjorleifsson, K. E., Eggertsson, H. P., Gudjonsson, S. A., *et al.* (2017). Whole genome characterization of sequence diversity of 15,220 icelanders. *Scientific data*, **4**, 170115.

Li, H. (2013). Aligning sequence reads, clone sequences and assembly contigs with bwa-mem. *arXiv preprint arXiv:1303.3997*.

Table 1. Breakdown of runtime analysis comparison, all times are given per sample.

	popSTR	popSTR2
samples	15,220	40,121
markers	880,355	5,401,401
polymorphic markers	380,261	1,636,021
Read selection	9.2 h	9.45 h
Sample slippage	0.25 h	0.065 h
Genotyping	0.35 h	2.19 h
CPU hours/1,000,000 markers	11.25	2.17

Table 2. Motif length distribution of kernel vs entire marker set

Motif length	Fraction of kernel	Fraction overall
1bp	48.2%	44.4%
2bp	23%	18.5%
3bp	4.5%	5.5%
4bp	12.8%	15.3%
5bp	6%	7.9%
6bp	5.5%	8.4%

Table 3. Comparison of hipSTR and popSTR2 on runtime and accuracy for chr21.

	CEU trio		deCODE trios	
	hipSTR	popSTR2	hipSTR	popSTR2
Runtime	57.48m	34.75m	9.70h	7.15h
Accuracy	99.6%	99.2%	99.6%	99.8%

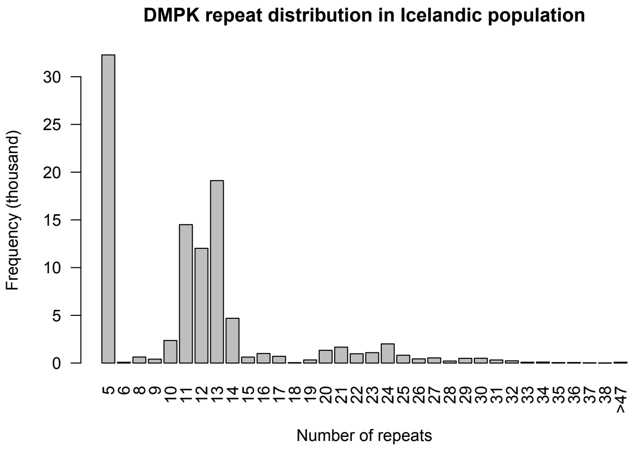

Fig. 1. Distribution of repeat numbers in Icelandic population at DMPK locus. Peaks at five repeats and 11-13 repeats match previously reported distributions in European populations.

Table 4. Disease-associated markers in panel and corresponding pathogenicity thresholds.

Location (HG38)	Gene	Disease	Pathogenicity threshold	OMIM link
chr1:149390803-149390842	NBPF19	Neuronal intranuclear inclusion disease	90	603472
chr2:176093058-176093099	HOXD13	Synpolydactyly 1	21	186000
chr2:190880873-190880920	GLS	Global developmental delay, progressive ataxia, and elevated glutamine	90	618412
chr3:63912685-63912716	ATXN7	Spinocerebellar ataxia 7	37	164500
chr3:129172577-129172659	CNBP	Myotonic dystrophy 2	50	602668
chr4:3074877-3074940	HTT	Huntington disease	36	143100
chr4:41745976-41746022	PHOX2B	Congenital central hypoventilation	25	209880
chr5:146878728-146878759	PPP2R2B	Spinocerebellar ataxia 12	55	604326
chr6:16327634-16327724	ATXN1	Spinocerebellar ataxia 1	39	164400
chr6:45422750-45422794	RUNX2	Cleidocranial dysplasia	27	119600
chr6:170561907-170562017	TBP	Spinocerebellar ataxia 17	47	607136
chr7:27199680-27199729	HOXA13	Hand-foot-genital syndrome	18	140000
chr8:104588965-104588999	LRP12	Oculopharyngodistal myopathy	93	164310
chr9:27573485-27573546	C9orf72	Amyotrophic lateral sclerosis	21	105550
chr9:69037285-69037304	FXN	Friedreich ataxia 1	200	229300
chr10:79826377-79826404	LOC642361/ NUTM2B-AS1	Oculopharyngeal myopathy with leukoencephalopathy	-	NatGen
chr12:6936717-6936775	ATN1	Dentatorubral pallidoluysian atrophy	49	125370
chr12:111598950-111599019	ATXN2	Spinocerebellar ataxia 2	35	183090
chr13:70139384-70139429	ATXN8OS	Spinocerebellar ataxia 8	111	608768
chr13:99985449-99985494	ZIC2	Holoprosencephaly-5	25	609637
chr14:23321472-23321492	PABPN1	Oculopharyngeal muscular dystrophy	12	164300
chr14:92071011-92071052	ATXN3	Spinocerebellar ataxia 3	55	109150
chr16:87604283-87604329	JPH3	Huntington disease-like-2	50	606438
chr18:55586154-55586229	TCF4	Corneal dystrophy	40	613267
chr19:13207859-13207898	CACNA1A	Spinocerebellar ataxia 6	20	183086
chr19:18786035-18786050	COMP	Multiple epiphyseal dysplasia	6	132400
chr19:45770205-45770266	DMPK	Myotonic dystrophy 1	50	160900
chr20:2652733-2652775	NOP56	Spinocerebellar ataxia 36	650	614153
chr21:43776445-43776479	CSTB	Myoclonic epilepsy of Unverricht and Lundborg	30	254800
chr22:45795355-45795424	ATXN10	Spinocerebellar ataxia 10	800	603516
chrX:67545318-67545383	AR	Kennedy disease	37	313200
